# Wildfire response to changing daily temperature extremes in California’s Sierra Nevada

**DOI:** 10.1126/sciadv.abe6417

**Published:** 2021-11-17

**Authors:** Aurora A. Gutierrez, Stijn Hantson, Baird Langenbrunner, Bin Chen, Yufang Jin, Michael L. Goulden, James T. Randerson

**Affiliations:** 1 Department of Earth System Science, University of California, Irvine, CA, USA.; 2 Geospatial Data Solutions Center, University of California, Irvine, CA, USA.; 3 Department of Land, Air and Water Resources, University of California, Davis, CA, USA.

## Abstract

Burned area has increased across California, especially in the Sierra Nevada range. Recent fires there have had devasting social, economic, and ecosystem impacts. To understand the consequences of new extremes in fire weather, here we quantify the sensitivity of wildfire occurrence and burned area in the Sierra Nevada to daily meteorological variables during 2001–2020. We find that the likelihood of fire occurrence increases nonlinearly with daily temperature during summer, with a 1°C increase yielding a 19 to 22% increase in risk. Area burned has a similar, nonlinear sensitivity, with 1°C of warming yielding a 22 to 25% increase in risk. Solely considering changes in summer daily temperatures from climate model projections, we estimate that by the 2040s, fire number will increase by 51 ± 32%, and burned area will increase by 59 ± 33%. These trends highlight the threat posed to fire management by hotter and drier summers.

## Teaser

High temperature extremes cause fire occurrence and burned area to increase nonlinearly in California’s Sierra Nevada.

## INTRODUCTION

Lightning-ignited wildfires have been prevalent in western North America and the Sierra Nevada ecoregion for millions of years, influencing the evolution of vegetation and wildlife (*1*–*3*). During the Holocene, fire management by indigenous peoples led to a fire regime with frequent and low intensity fires, often with a return interval of less than 20 years (*1*) and yielding outcomes that improved the efficiency of resource gathering and other ecosystem services (*4*, *5*). This fire regime was altered by the loss of indigenous peoples caused by waves of Spanish, Mexican, and U.S. settlers and in the mid-19th century by the California Gold Rush, which contributed to deforestation and heavy livestock grazing across the region (*2*). Early and mid-20th century fire suppression policies also shaped the fire regime, causing vegetation composition and structure to shift over time (*6*, *7*). There is a general consensus that these changes contributed to a new fire regime of infrequent, high-intensity fires that was prevalent during the middle and latter half of the 20th century (*4*, *8*–*10*). During the 21st century, changing climate is expected to further modify ecosystem structure and function, and a key challenge is to understand how the fire regime of the Sierra Nevada will evolve in response to warming.

Over the past few decades, fire occurrence and burned area have increased considerably in the Sierra Nevada ecoregion (*3*). An improved attribution of the recent increase in burned area is needed for better predictions of future fire activity and for the design of forest management strategies but remains challenging given the wide range of possible drivers and the interactions among them. Fire suppression and land-use change have been considered primary drivers as they have led to denser, more flammable vegetation with a more connected landscape for fire spread (*8*, *11*). Concurrently, population growth and housing development have increased, particularly in lower elevation foothills, potentially increasing the frequency of ignition in and around the wildland-urban interface (*12*–*14*). A third key potential driver is climate change, with observations providing evidence of hotter, drier conditions during summer and a longer fire season (*12*, *15*–*17*). Higher temperatures and increases in vapor pressure deficit (VPD) can influence fire risk by drying fuels, thus making them more flammable and prone to ignition (*10*, *17*, *18*). Once ignited, fires in areas with drier fuels can spread more rapidly, making them difficult to contain (*19*).

Disentangling the relative contribution of these drivers poses a formidable challenge. Previous studies have relied on monthly or annual burned area statistics when considering the influence of climate on wildfire trends across the western United States. However, fire ignition and fire spread are often driven by short-term meteorological conditions, and less work has explored how heat waves and other daily weather extremes have affected fire trends. Here, we quantify the influence of temperature variability on fire occurrence and burned area by combining time series of daily meteorological conditions, fire occurrence, and daily burned area derived from satellite imagery. We then use the resulting statistical relationships to reconstruct past and project future changes in fire number and burned area during summer in the Sierra Nevada ecoregion.

## RESULTS

### Sensitivity of fire activity to daily temperature extremes

**Fig. 1.**
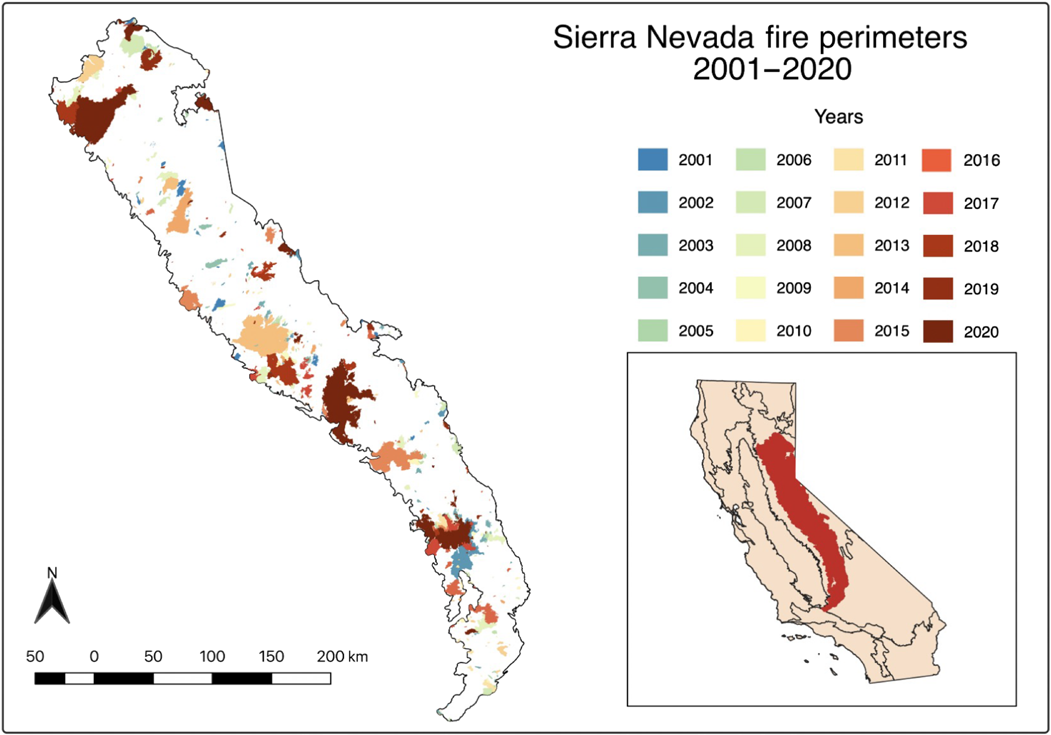
The location and perimeters of summer fires in California’s Sierra Nevada ecoregion from 2001 through 2020. All fires with a final size greater than 40.5 ha (100 acres) are shown by year using geospatial data from the FRAP (*20*) database developed by the California Department of Forestry and Fire Protection. The Sierra Nevada level 3 ecoregion within California is shown in the inset panel.

During 2001–2020, there were 441 fires larger than 100 acres (0.405 km^2^) in the Sierra Nevada (*20*). Most of the fires occurred during the hot and dry summer, with the 4-month period from June 1st through September 30th accounting for more than 86% of the annual number of fires and 94% of the total annual burned area according to data from the California Department of Forestry and Fire Protection’s Fire and Resource Assessment Program (FRAP; fig. S1, A and B) (*20*). The summer fires were widely distributed across the Sierra Nevada, covering 23% of the area of the domain (Fig. 1). Both the number of fires and burned area associated with summer fires varied considerably from year to year (fig. S1, C and D). Daily 500-m burned area observations derived from NASA’s Moderate Resolution Imaging Spectroradiometer (MODIS) instruments on the Aqua and Terra satellites (*21*) show good agreement with the FRAP fire perimeters on both seasonal and interannual time scales (fig. S1, B and D). Analysis of the MODIS observations revealed that one or more wildfires were burning on 1284 summer days during 2001–2020, or 53% of the time, with fires observed in about 4.9 × 10^4^ individual 500-m MODIS pixels.

**Fig. 2.**
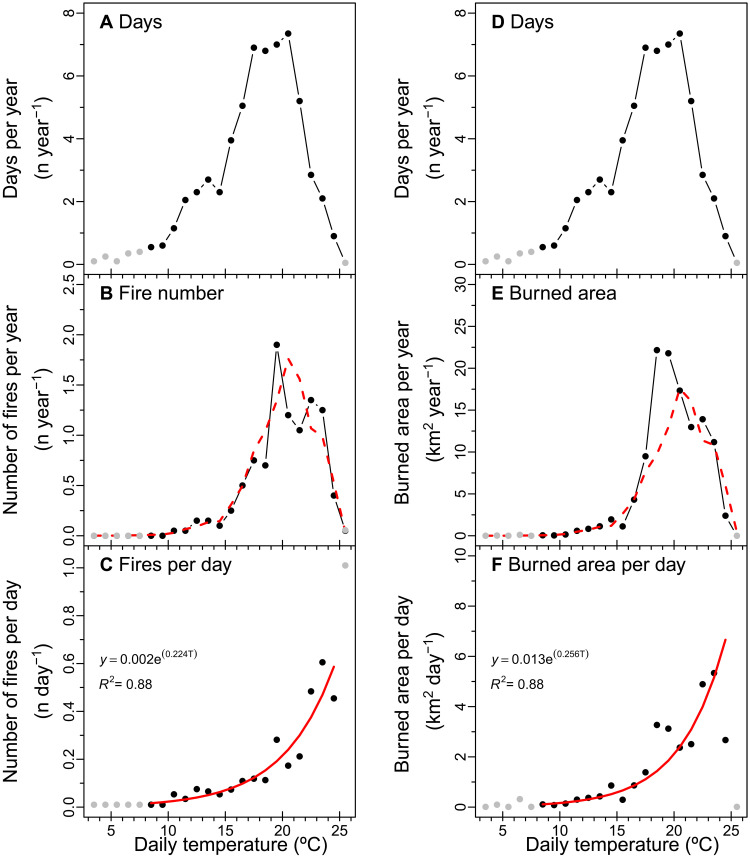
Sensitivity of fire number (left panels) and burned area (right panels) to daily temperature for June to July. (**A**) and (**D**) show the mean number of days within each 1°C temperature interval during 2001–2020. These panels are identical and are shown twice to help with vertical visual comparison with fire number and burned area. (**B**) and (**E**) show the annual number of fires and burned area for the same daily temperature intervals. Daily temperatures are from PRISM and represent the region-wide mean for the Sierra Nevada level 3 ecoregion. Fire number is from FRAP, and burned area is from MODIS. (**C**) and (**F**) show the number of fires and burned area per day as a function of daily temperature. These distributions were derived by dividing the observations in the middle panel by the observations in the top panel. The red lines in the bottom panels show nonlinear model fits. The red dashed lines in the middle panels show estimated distribution of fire number and burned area obtained by combining the models from the bottom panels with the number of days PDF from the top panels. Daily temperature intervals with less than 10 of the total number of days during 2001–2020 are shown in gray and were not used to derive the model form or parameters. The relationships in (C) and (F) were highly significant (*P* < 0.01) when assessed using linear regression on the log-transformed fire variables.

**Fig. 3.**
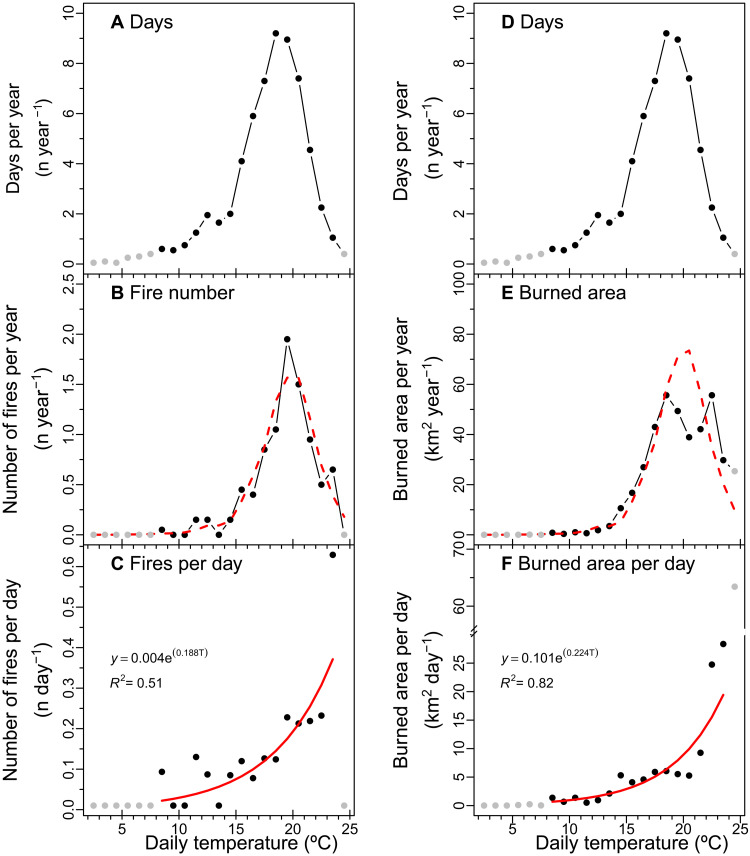
PDFs for the number of days, fire number, and burned area as a function of daily mean temperature during 2001–2020 for the August to September interval. The information in this figure was constructed in an identical way to that shown in Fig. 2 but describes climate and fire observations during August and September rather than June and July. The relationships in (C) and (F) were highly significant (*P* < 0.01) when assessed using linear regression on the log-transformed fire variables.

To assess how day-to-day temperature variability influences the probability of fire occurrence and region-wide burned area, we combined a daily, region-wide meteorological record from the Parameter-elevation Regressions on Independent Slopes Models (PRISM) Climate Group (*22*) with data on the timing of fires from FRAP (*20*) and of the record of daily burned area from MODIS (*21*). We first separated the summer into two periods (June to July and August to September) to reduce variation in temperature caused by seasonal trends. We then generated probability distribution functions (PDFs) of the number of days and each fire variable for 1°C daily temperature bins. The PDF of June to July daily temperatures shows a peak between 17° and 20°C (Fig. 2, A and D). Compared to the PDF for summer days, the PDFs for fire number and daily burned area are visually shifted to the right along the temperature axis (Fig. 2, B and E). We then estimated the sensitivity of fire activity to daily temperature in a two-step process. First, we divided the PDFs of fire number or burned area in the middle panels by the PDF of temperature occurrence shown in the top panels. The resulting distributions illustrate the relationships between temperature on a single day and the probability of fire activity (Fig. 2, C and F). Second, we fit exponential curves through these distributions. The resulting relationships [red lines in Fig. 2 (C and F)] provide a means for projecting the impact of changing summer temperatures on daily fire occurrence and burned area during periods outside of the satellite era. A comparable analysis for the August to September period is shown in Fig. 3.

Our analysis of the PDFs revealed that the probability of wildfire occurrence has a strong, highly significant positive relationship with daily temperature during both June to July and August to September summer intervals (*P* < 0.001; Figs. 2C and 3C). Using *R*^2^ as a goodness of fit indicator of model performance, a two-parameter exponential model was better at describing the relationship between daily temperature and fire occurrence than a linear model with the same degrees of freedom (table S1). The probability of fire occurrence increases disproportionately on hotter days in these nonlinear models. As a measure of the sensitivity of wildfire occurrence to changes in daily temperature, we computed the slope of the relationship at the mean daily temperature for each summer interval. These relationships indicated that a 1°C increase in daily temperature increases the probability of a fire start by 22.3 ± 0.7% during June to July and 18.8 ± 1.5% during August to September (Table 1).

**Table 1.** Sensitivity of fire number and burned area to daily temperature relative to the 2001–2020 mean temperature. Temperature sensitivity estimates of fire number and burned area were computed from the relative slope of the nonlinear models in Figs. 2 and 3 at the mean daily temperature for each summer interval (17.7° ± 3.8°C for June to July and 17.8° ± 3.4°C for August to September). The uncertainties were estimated using a jackknife approach, denotated with a ± for 1 SD.

**Fire variable**	**Sensitivity factor to daily temperature**	
	**June–July**	**August–September**
Fire number	22.3 ± 0.7% per degree Celsius	18.8 ± 1.5% per degree Celsius
Burned area	25.5 ± 0.7% per degree Celsius	22.3 ± 0.8% per degree Celsius

Daily burned area also has a strong, positive relationship with daily temperature during summer (*P* < 0.001; Figs. 2F and 3F). A 1°C increase in daily temperature increases burned area by about 25.5 ± 0.7% during June to July and 22.3 ± 0.8% during August to September.

Sorting fire activity by percentiles of daily temperature during 2001–2020 also highlights the importance of extremes (fig. S2). The warmest 10% of days accounts for 24 to 31% of the total number of fires across the two summer periods and 23 to 33% of the total burned area. Similarly, the warmest 25% of days accounts for 43 to 49% of the total number of fires and 42 to 47% of the total burned area.

We hypothesize that the strong positive relationships between daily temperature and fire activity are a consequence of warmer days drying fine fuels, which, in turn, increases the likelihood of successful ignition, fire escape from human control, and rate of fire spread. In a sensitivity study, we replaced daily temperature with daily maximum VPD as the driver variable. This analysis also provides evidence for a nonlinear relationship between VPD and wildfire activity (fig. S3), which supports a drying mechanism and is consistent with past analyses evaluating VPD as a driver at monthly and seasonal time scales (*3*, *16*, *23*). We computed sensitivity factors for daily maximum VPD following the same approach described above for temperature. For June to July, a 1-hPa increase in VPD yields a 10.0 ± 0.6% increase for fire number and an 11.6 ± 0.5% increase for burned area. For August to September, a 1-hPa increase yields an 8.3 ± 1.0% increase for fire number and 8.9 ± 0.9% increase for burned area. The VPD-based sensitivities are broadly consistent with those reported above for temperature, given that VPD increases at a rate of about 1.9 hPa per degree Celsius for the Sierra Nevada ecoregion during summer (computed at mean summer temperature of 17.7°C) (fig. S4). Further examination of the relationship between daily temperature and daily maximum VPD in fig. S4 shows that temperature has a dominant role in structuring day-to-day variability in VPD during summer.

### The role of summer temperature in regulating past and future fire activity

**Fig. 4.**
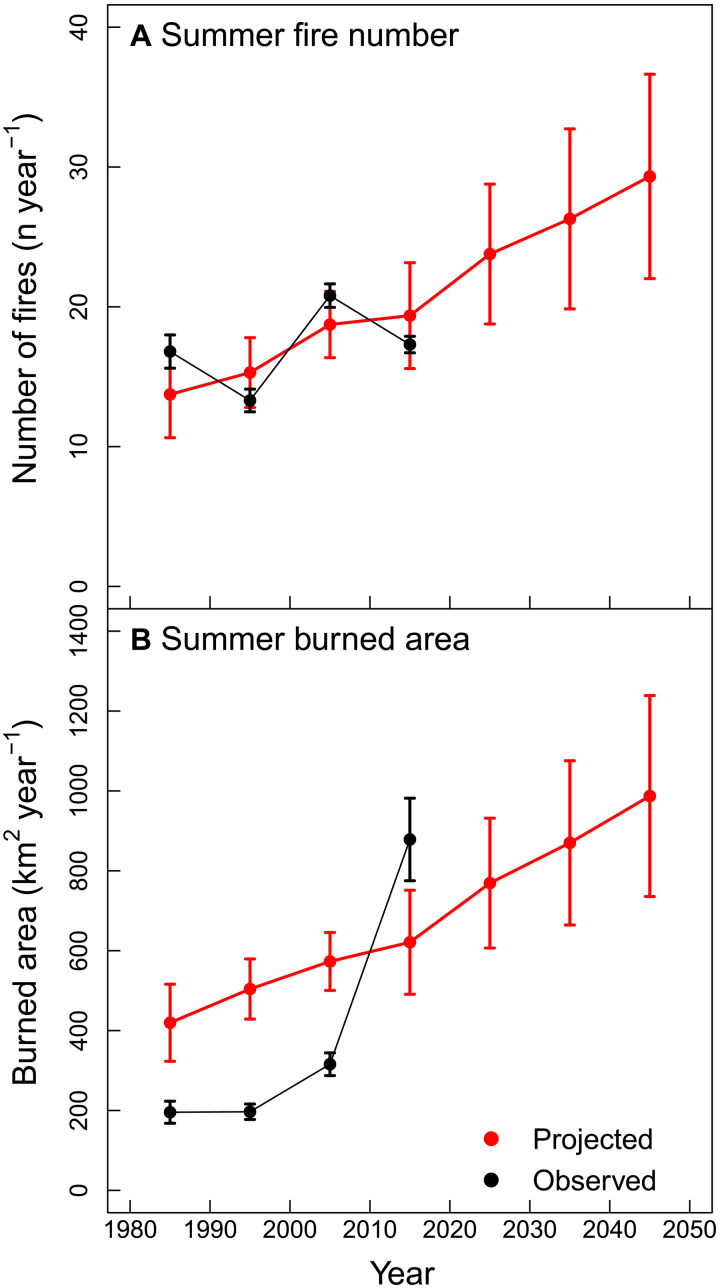
Projected increases in fire number and burned area from summer warming for the Sierra Nevada ecoregion in California. The observations of summer fire (**A**) number and burned area (**B**) from the FRAP dataset are shown in black. The model estimates in red show the impact of changing summer daily air temperature on fire activity using the relationships derived from fire observations during 2001–2020. The uncertainties were estimated using a jackknife approach, denotated with a ± for 1 SD. Projections of the impact of changing summer temperature on fire activity from the 1980s through 2010s were obtained using observations from PRISM. Future projections of the impact of changing daily temperatures on fire activity from the 2010s through the 2040s were obtained using earth system model simulations from CESM1 LENS. Summer temperatures explains only about 30% of the observed trend in burned area in the observations, indicating that other climate and nonclimate processes are important in explaining observed increases over the past four decades.

**Fig. 5.**
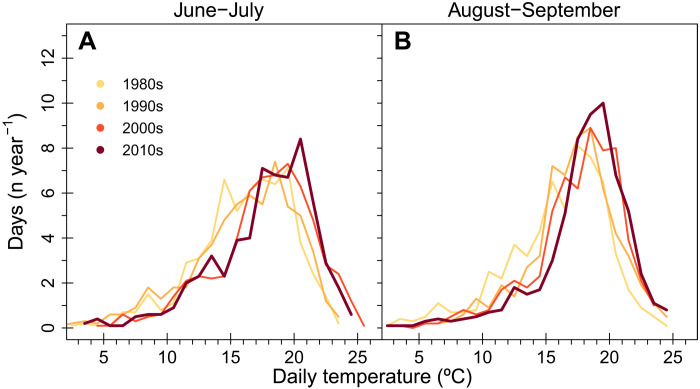
PDFs of daily temperature by decade from the 1980s through the 2010s. The PDFs were created from PRISM climate observations during June to July in (**A**) and during August to September in (**B**).

Between the 1980s and the 2010s, summer burned area increased by more than threefold in the Sierra Nevada ecoregion (Fig. 4 and fig. S5). Over this period, the PDF of daily summer temperature shifted considerably (Fig. 5), resulting in a summer mean in the 2010s (17.8° ± 3.6°C) that was significantly higher (Student’s *t* test; *P* < 0.001) than in the 1980s (16.0° ± 3.8°C). To estimate the contribution of the changing summer temperature to trends in fire activity, we applied the fire-temperature relationships shown in Figs. 2 and 3 to the PDFs of daily temperature observations for each consecutive decade from PRISM shown in Fig. 5. From this analysis, we estimated that warming summers increased the number of fires by 41 ± 30% and burned area by 48 ± 31% over the past four decades (Table 2). The predicted increase in fire occurrence is consistent with the observations, although there is no significant trend in the observations as a consequence of high levels of decade-to-decade variability. For burned area, our estimate of the increase in total area attributable to warming surface air temperature from the 1980s to the 2010s (202 ± 162 km^2^ year^−1^) represents about 30% of the observed increase of 683 km^2^ year^−1^ (Fig. 4). Our analysis provides evidence that summer warming contributes to some but not all of the observed increases in burned area, implying that additional meteorological and ecological drivers must contribute to the full magnitude of the observed trend.

**Table 2.** Model estimates of changes in fire number and burned area caused by summer climate warming in the Sierra Nevada ecoregion. Percent change for each decade is reported relative to a baseline of the 1980s. Fire observations from FRAP are also shown along with mean surface air temperature from PRISM for 1980s through 2010s. Future estimates of summer temperature, denoted with a * are from the CESM1 LENS project for the RCP85 scenario (*24*). The uncertainties were estimated using a jackknife approach, denotated with a ± for 1 SD.

**Time period**	**Summer mean** **temperature** **(°C)**	**Fire number**			**Burned area**		
		**n year^−1^**	**Δ%**	**Obs.** **n year^−1^**	**km^2^ year^−1^**	**Δ%**	**Obs.** **km^2^ year^−1^**
1981–1990	16.0 ± 3.8	13.7 ± 3.1	0	16.8 ± 1.2	419.6 ± 96.5	0	195.5 ± 27.6
1991–2000	16.6 ± 3.8	15.3 ± 2.5	11	13.3 ± 0.8	504.0 ± 75.3	20	196.7 ± 19.4
2001–2010	17.6 ± 3.6	18.7 ± 2.4	36	20.8 ± 0.8	573.0 ± 72.5	37	315.7 ± 28.5
2011–2020	17.8 ± 3.6	19.4 ± 3.8	41	17.3 ± 0.6	621.1 ± 130.2	48	878.4 ± 103.3
2021–2030*	18.6 ± 3.9	23.8 ± 5.0	73		769.2 ± 162.6	83	
2031–2040*	19.1 ± 3.9	26.3 ± 6.4	91		869.9 ± 205.6	107	
2041–2050*	19.8 ± 3.9	29.3 ± 7.3	114		987.2 ± 251.6	135	

To assess the impact of expected future increases in summer temperatures on fire activity in the Sierra Nevada, we combined our temperature-fire relationships derived from observations with simulations from the Community Earth System Model version 1 (CESM1) large ensemble (LENS) (*24*). Temperatures from LENS were bias-corrected using PRISM observations, and we limited our projections to the next three decades for two reasons. First, fire and drought feedbacks on vegetation composition and structure are likely to be less important over a period of a few decades as compared to the integrated effects of these feedbacks by 2100. Second, over a period of a few decades, much of the expected climate warming is already “in the pipeline” as a consequence of adjustments to past forcing (*25*). This means that climate projections from different emission scenarios do not considerably diverge (*26*), and we can take advantage of LENS for representative concentration pathway 8.5 (RCP8.5) to reduce the sensitivity of our projections to internal climate variability (*24*, *27*). By the 2040s, the mean increase in summer temperatures in LENS is 2.0°C across the Sierra Nevada relative to the 2010s (fig. S6 and Table 2) and is similar to other Coupled Model Intercomparison Project Phase 5 (CMIP5) projections (fig. S7). Drawing on the temperature-fire relationships we derived in the “Sensitivity of fire activity to daily temperature extremes” section, we estimate a corresponding increase in the number of summer fires of 51 ± 32% and burned area of 59 ± 33% compared to the 2010s (Fig. 4 and Table 2).

The increase in future fire activity from our analysis is driven by both an increase in mean temperature, with the PDF of daily temperatures shifting to the right (fig. S6, A and D) and a widening of the PDF of temperature within individual LENS ensemble members (fig. S6, C and F). The increase in daily temperature variability during summer is robust across the different summer intervals (June to July and August to September) and across different LENS ensemble members. The future increases in daily temperature variability amplify fire risk beyond that expected solely from changes in the mean because of the nonlinear sensitivity of fire activity to temperature. In a sensitivity analysis, we used the daily maximum VPD-wildfire relationships shown in fig. S3 as the basis for future projections. With VPD, we estimate a 44 ± 31% increase in the number of summer fires and a 42 ± 31% increase in burned area compared to the 2010s (fig. S8). These projections have a similar magnitude to those based on daily summer temperature and are in broad agreement given the uncertainty estimates.

## DISCUSSION

### Extremes in daily temperature as a driver of fire activity

Daily temperature plays a significant role in shaping fire behavior during the summer in the Sierra Nevada ecoregion. Most previous analyses have emphasized seasonal mean temperature as the primary driver variable (*17*, *28*, *29*), whereas, here, we separated the role of daily temperature from other drivers of fire risk and showed that the hottest days during summer have a disproportionate and nonlinear effect on fire activity.

The importance of daily temperature as a driver points toward a growing fire threat from climate change and possible targets for managing this threat. From a human health perspective, expected increases in summer heat waves with climate warming (*30*, *31*) will contribute to more hospital visits and higher mortality (*32*). Our findings indicate that these heat waves will also simultaneously reduce air quality in California and the western United States as a consequence of elevated burning. While several recent papers have isolated western wildfire aerosol impacts on human health and economic sectors (*33*, *34*), our work suggests that further research is needed to understand the covariance between heatwaves, fires, and air quality on a daily time scale and the degree to which interactions between them amplify health risks for vulnerable populations (and firefighters).

From a management perspective, the relationships that emerge from our analysis of daily variability may help identify priorities for limiting the ignition of large and destructive wildfires. While past work in California has explored the relationship between climate and fire number in the western United States (*29*, *35*, *36*), most of these analyses have focused on seasonally averaged conditions, and this body of literature is generally less developed than analysis of the climate–burned area relationship [e.g., (*3*)]. Within the fire science community, a strong distinction is made between lightning and human-caused ignition (*37*), with human-caused ignitions often viewed as possible once a minimum set of environmental conditions (fuel availability and dryness) are met. This basic assumption has also been integrated within several global fire models (*38*). Here, we show that the likelihood of an ignition of a large wildfire has a nonlinear relationship with daily temperature in Sierra Nevada forests, with no apparent saturation of risk on the very hottest of days (Figs. 2 and 3). This means that current estimates of past and future fire occurrence relationship may underestimate climate-driven changes in ignition and initial fire spread. In addition, the relationships we describe may be combined with weather forecasts to improve forecasts of wildfire risk. Specifically, our work suggests that there may be value in targeting temperature extremes, even outside periods of existing red flag conditions (*9*, *30*), for additional fire prevention measures.

### Underlying mechanisms

The strong positive effect of daily temperature on fire occurrence we observed is likely mediated, in part, by a relationship between temperature and the likelihood of a fire escaping initial human control. For example, the accidental ignition of one of the largest fires in California’s history, the Ranch Fire (which later merged into the Mendocino Complex Fire), was triggered by sparks from a rancher’s hammer as he drove a metal stake into the ground to plug a wasp’s nest (*39*). This sort of activity poses limited fire risk under normal conditions. However, under the hot and dry conditions at the time, the fire rapidly spread through nearby grasses and became uncontrollable, despite the rancher’s attempts to extinguish it. There are many similar anecdotal reports of higher risk of fire incidence during heatwaves, and our analysis shows that it is possible to quantify the form of this relationship. Mechanistically, high daily temperature, which dries fine fuels, likely increases the probability of large wildfire occurrence in three different ways. First, the lower fuel moisture and thus higher flammability on a hot day increases the efficacy of ignition sources in triggering flaming combustion (e.g., the likelihood of the metal sparks creating a flame in the above example). Second, once flaming combustion has been initiated, the lower fuel moisture enables more rapid spread, making the initial containment more difficult by individuals at the location of ignition and fire personnel responding to the event. Third, the higher initial rate of fire spread also may increase the resilience of a wildfire to subsequent periods of less favorable weather as a consequence of a longer and more heterogeneous active fire line. The influence of climate change on the processes regulating fire escape and initial rates of spread have not been systematically explored, and more work is needed to develop more accurate models and projections of future change.

The nonlinear sensitivity of fire occurrence to daily climate identified here suggests that that ignition and initial escape processes may play a key role in contributing to the exponential climate–burned area relationships observed in past work on monthly and seasonal time scales (*3*, *40*–*42*). We initially hypothesized that daily burned area in the Sierra Nevada would have a stronger positive relationship with daily temperature than fire occurrence. Our rationale was that hot days would lead to more fire starts and more rapid expansion of existing active wildfires. We thought that together, these two processes would amplify the sensitivity of burned area to temperature. The findings in Figs. 2 and 3 and Table 1 indicate that, contrary to our hypothesis, burned area has a similar sensitivity to daily temperatures as fire occurrence. We believe that a likely explanation is tied to the duration of summer heatwaves. Periods of extreme high temperature increase the probability of fire occurrence (Figs. 2 and 3) but often last for a few days. During this time, many of the fires that are initially ignited during the heatwave may grow into larger wildland fires that require weeks or months before they are fully contained. Examples of these longer duration (and larger) wildfires include the Rim fire that burned in 2013 for about 35 days, the Rough Fire that burned in 2015 for 54 days, and the Creek Fire that burned in 2020 for 42 days. Over the lifetime of these fires, daily temperatures may decline considerably from levels that occur during the initial period of extreme fire weather responsible for their start, yet because fire perimeters can grow exponentially over time, daily burned area remains high, and the fires remain difficult to contain. This may have the effect of lowering the effective sensitivity of the burned area–daily temperature relationship.

### Attribution of recent burned area trends

Summer fires in the Sierra Nevada forests have increased as a consequence of exceptionally warm days. Our estimate of the contribution of warming to observed trends is lower than other recent assessments. We estimate that about 30% of the total increase in burned area from the 1980s through the 2010s can be attributed directly to increasing summer air temperature. In contrast, Williams *et al.* (*3*) concludes that nearly all of the increase in California burned area can be attributed to climate warming from the 1970s through 2018. The difference in estimates between the two studies can be traced, in part, to contrasting methods and time periods of analysis. Anthropogenic climate change does not solely influence summer air temperatures but also changes snowpack (*43*–*45*), lightning frequency (*46*), summer atmospheric moisture levels (*47*), and the intensity and severity of drought (*48*). Earlier snowmelt, for example, may lead to more frequent wildfires by extending the dry season (*17*, *49*), making fuels more flammable in midsummer. Williams *et al.*’s use of seasonally integrated variables likely accounts for many of these additional processes, thus yielding a higher estimate of the contribution of anthropogenic climate change. For the wider domain of the western United States, Abatzoglou and Williams (*28*) find that climate change nearly doubled burned area during 1984–2015; again, the seasonally averaged fuel aridity metrics used in their analysis likely integrate across a broader set of climate change processes. To further reduce uncertainties associated with the impact of climate change on recent trends, an important next step is to quantify covariances among different driver variables to better understand and control interactions on multiple time scales.

Other processes not related to climate have considerably evolved over the past four decades in the Sierra Nevada, influencing the spatial pattern of fuels and ignition risk (*50*). Thus, some of the difference between the red and black lines shown in Fig. 4 may also reflect concurrent changes in land use, vegetation composition, ecosystem management, and resources available for fire suppression.

### Future projections of wildfire response to changes in summer temperature

We estimated that increasing daily summer temperature extremes will increase the number of fires by 51 ± 32% and burned area by 59 ± 33%, through the 2040s relative to a 2011–2020 baseline (Fig. 4 and Table 2). Our work supports the conclusion that considerable potential exists for an increase in fire activity as a consequence of climate warming in the absence of changes in fire and ecosystem management (*12*, *17*, *18*, *29*, *51*, *52*). We emphasize that our analysis of future fire activity considers only one factor (summer daily temperature) and that other climate change impacts on ecosystem function and fire dynamics are expected (*3*, *17*, *28*). These impacts may dampen or strengthen the projected changes in fire activity reported here and include changes in fire suppression, vegetation dynamics, and the strength of institutions responsible for land management. Important potential negative feedbacks include (i) changes in human behavior (and adaptation) in response to increasingly extreme summer heatwaves (*53*) and (ii) the influence of increasing levels of burning on vegetation composition and fuels in the Sierra Nevada ecoregion. Westerling (*54*), for example, accounted for changing vegetation dynamics, human population, housing development, land management responsibility, fuels management, and multiple climate and hydrologic variables from two future climate scenarios when developing wildfire scenarios for California’s Fourth Climate Change Assessment. Westerling’s analysis reveals that aggressive fuel management has the potential to reduce the magnitude of future increases in burned area driven by climate, although more work is needed using observations to understand how different fuel treatments influence fire behavior, including rates of fire spread, especially during periods with extreme fire weather.

Our findings have the potential to inform more comprehensive assessments of future fire dynamics. Our analysis suggests, for example, that new fire starts are highly sensitive to daily variation in surface air temperature and VPD, implicating fine fuel moisture status as having a key role in regulating whether a fire escapes initial containment. This information may inform the design of both mechanistic and statistical models of fire occurrence. Further, our work suggests that increasing variability of summer weather on daily time scales (fig. S6) poses an additional, inadequately understood risk for wildfire management.

By combining daily weather and fire time series, we examined how fine-scale temporal variability in surface air temperature during summer influences fire occurrence and burned area in the Sierra Nevada ecoregion. Our analysis shows that high temperature extremes have a disproportionate effect on fire activity, likely as a consequence of fine fuel drying. The strong, positive, and nonlinear relationship between daily temperature and fire occurrence we observe indicates that fire risk does not saturate during periods of extreme fire weather. Using this information together with future projections of summer temperature from an ensemble of earth system model simulations, we estimate that burned area will increase by over 50% by the 2040s. Our estimate is unique from other projections of fire in the western United States because it quantifies climate change impacts arising primarily from changes in the mean and variance of summer daily temperature. The form of the temperature–fire activity relationships we report may be useful for informing the design of more effective fire danger indices and for building more mechanistic representation of ignition and fire escape processes in fire models. A key direction for future research is to further analyze the daily satellite observations of fire to understand how new extremes in summer temperature will influence rates of spread and burn severity, since these two aspects of fire often regulate the magnitude of ecological and economic damage.

## MATERIALS AND METHODS

### Burned area and climate datasets

We obtained fire perimeter observations from the FRAP (version 20_1, 20) of the California Department of Forestry and Fire Protection (*20*). We included all fires that were within the Sierra Nevada level III ecoregion boundary defined by U.S. Environmental Protection Agency (EPA) (*55*). We used a minimum fire size threshold of a 40.47 ha (100 acres) in our time series to ensure temporal continuity. A minimum fire size threshold helped to reduce discrepancies caused by reporting requirements thresholds that have evolved over time (*51*). Applying this threshold, we identified 381 fires that were ignited in summer (June to September) during 2001–2020 (Fig. 1 and fig. S1). We used the start date of these individual fires from FRAP to assess the impact of daily temperature variability on the probability of fire occurrence.

We used the NASA’s MODIS burned area product MCD64A1 Collection 6 (*21*) to provide daily burned area at 500-m resolution for the entire study period except during 2020. Because of sensor problems with the Aqua satellite, burned area for summer 2020 was not available when we performed the analysis. Therefore, we used 2020 Visible and Infrared Imaging Radiometer (VIIRS) active fire detections, rasterized at 500-m resolution, to identify the day of burning with available fire perimeter polygons. We assigned the earliest day of detection within each pixel and used an inverse distance-weighted interpolation algorithm using the five nearest values to estimate date of burn for areas inside the burned area perimeter polygons that did not contain active fire detections. Reference burned area perimeters for the summer 2020 were obtained from the National Incident Feature Service through the California State GeoPortal.

Previous independent validation of the MCD64A1 product with VIIRS thermal fire detections has shown that 54% of day of burn estimates are within ±1 day of the peak in VIIRS detections for forests, and 80% are within ±1 day of the peak in VIIRS detections for shrublands (*56*). Over the period 2001–2020 (20 years), we identified that an area of 10,478 km^2^ burned during summer in the Sierra Nevada from the MCD64A1 product. This was about 12% less than the estimate from FRAP and consistent with the likelihood that unburned islands exist within the final FRAP perimeters. On a year-to-year basis, MODIS and FRAP coincide well (fig. S1). Burning during June through September accounted for about 92% of the annual mean burned area from MODIS and 94% from FRAP (fig. S1). We generated a daily time series of the region-wide burned area to compare with the meteorological observations by taking the sum of all 500-m MODIS burned area pixels identified for an individual day within the Sierra Nevada ecoregion. Over the 20 years of this time series, 1284 days (53% of the June to September period) had some level of burning in the Sierra Nevada. On many days, the region-wide burned area had contributions from the expansion of multiple fires distributed throughout the domain. Over 2001–2020, our time series of region-wide daily burned area for summer integrated 48,812 individual 500-m burned area pixels from the MODIS product.

For meteorological conditions from 1981 through 2020, we used daily gridded data from the PRISM Climate Group (www.prism.oregonstate.edu/recent/). PRISM uses available weather station data and a climatically aided interpolation to provide a comprehensive temporal and spatial interpolation across the region at a daily 4-km spatial resolution (*57*). For each day within the Sierra Nevada level III ecoregion, we computed an area-weighted mean of PRISM daily surface air temperature and daily maximum VPD using the individual 4-km grid cells.

To quantify how future climate changes may affect the burned area, we extracted daily 2m surface air temperature (T2M) from the CESM1 LENS (www.cesm.ucar.edu/projects/community-projects/LENS/data-sets.html) from the 1980s to the 2040s (*24*). Each of the 40 simulations in this ensemble had slightly different initial conditions in the atmosphere starting in 1920 and diverged over time from the effects of internal climate variability. All of the simulations were forced by the same greenhouse gases and aerosol time series from the representative concentration pathway 8.5 (*58*).

To assess the representativeness of LENS relative to other climate projections, we compared LENS temperature trends with other CMIP5 models processed using the multivariate adaptive constructed analogs (MACA) approach (*59*) and accessed at www.climatologylab.org/maca.html. We bias-corrected each LENS simulation to the mean of the bias-corrected CMIP5 RCP85 simulations during 2006–2020. This analysis shows that LENS is broadly representative of the mean trend and variability of the CMIP5 models for the Sierra Nevada level 3 ecoregion during the 21st century (fig. S7). We calculated an area-weighted daily mean for each LENS realization across the Sierra Nevada ecoregion, accounting for the fraction of each 1° × 1° grid cell that was within the ecoregion perimeter. We bias-corrected each of the 40 LENS simulations so that mean daily temperatures for each summer interval (June to July and August to September) were the same for PRISM and LENS during 2006–2020. We did not adjust the variance of the LENS temperature time series because SDs in daily temperature from the LENS ensemble members matched the daily observations from MACA-processed CMIP5 models for the contemporary period reasonably well (fig. S7).

### Data analysis

To investigate how daily temperature influences both fire ignition and region-wide burned area in the Sierra Nevada, we combined the daily, region-wide weather time series with the start date and burned area time series. Our approach for deriving the distribution of fire number per day or burned area per day as a function of daily temperature is described in the “Sensitivity of fire activity to daily temperature extremes” section (Results). Our approach assumes that the mean temperature observation from PRISM on each day is given equal weight in creating the daily temperature—wildfire relationships are shown in Figs. 2 and 3 and that each daily temperature observation is independent of other observations. An important next step is to assess how the duration of heat waves and the ordering temperature anomalies among individual days influences fire risk.

To model the probability of fire occurrence or burned area each day as a function of daily temperature for historical or future projections, we fit an exponential function to the distributions in the bottom panels of Figs. 2 and 3. To construct these functions, we only used points in the distribution from temperature bins for 10 or more observations were available (out of a total of over 1200 days in each 2-month summer period spanning 2001–2020). We imposed this threshold to avoid low probability (and undersampled) events having a disproportionate effect on the shape of the modeled function. We performed a log transformation of each fire variable (fire number and burned area) and then performed a linear fit, to obtain the functional forms of the two parameter exponential relationships shown in Figs. 2 and 3. We assessed significance of the relationship using the *P* value obtained from the linear regression (table S1). We further report the relative slope of the exponential functions at the mean temperature of each summer interval as sensitivity factors with units of percent change in the number of fires or burned area for a 1°C increase in daily temperature.

For VPD–fire activity relationships, we created PDF of the distributions of maximum daily VPD at 5-hPa increments using observations from PRISM. We then conducted a similar analysis to that described above for temperature, excluding bins with less than 10 individual daily observations. For our VPD–fire number relationships, we used a three-parameter exponential relationship (including an offset) to reduce structural biases in the residuals.

We used the modeled temperature-fire functions to estimate the impact of climate change on past and future fire number and burned area. We limited our projections of climate change impacts to four decades before the present (the 1980s) and three decades into the future (2040s). This was done to limit the impacts of potential feedbacks from changing fire and drought regimes on fuel amount and flammability and the longer-term effects of changing management. For the historical era, we estimated the impact of warming since the 1980s using the daily surface air temperature time series from PRISM. For each decade, we generated a PDF of the number of days in 1°C temperature bins during each seasonal period (Fig. 5). We then applied the functions of fire number or burned area per day as a function of daily temperature derived from the 2001–2020 period when we had coincident FRAP, MODIS, and PRISM observations.

To project future changes in fire caused by climate change through the 2040s, we took a similar approach, using the bias-corrected CESM1 LENS time series to generate temperature and VPD PDFs for the decades of the 2020s, 2030s, and 2040s. We developed the bias corrections by matching the means for each summer period from LENS during the 2006–2020 period with the observations from PRISM. To develop conservative projections of future change with the nonlinear functions, we fixed potential rates of fire activity above the valid set of contemporary observations (shown with the black dots in Figs. 2 and 3 and fig. S3) at constant maximum fire levels observed during the satellite observations.

### Error analysis

We used a jackknife approach to estimate uncertainties associated with the sensitivity of the modeled relationships describing fire probability as a function of daily temperature or VPD to variability in the fire observations. In our approach, we sequentially removed one of the data bins from each PDF, in a leave-one-out strategy. We then fit a new model to the remaining observations. The ensemble of relationships derived from this approach was used to estimate uncertainties in the sensitivity factors (Table 1) and in the estimates of climate contributions to past and future wildfire activity driven by changes in summer temperature (Table 2).

Our past and future estimates of fire activity were performed separately for the June to July and August to September intervals, to be consistent with the methodology we used during the satellite era. When combining the two intervals to create summer-wide estimates in Table 2 and Fig. 4, we added the SDs together in quadrature, assuming the contributions from these two different intervals were independent. For the future projections, additional uncertainty was originated from performing the jackknife procedure individually on each of the 40 model ensemble members from CESM1 LENS. In a final step, we calculated the mean value and SD across all 40 of the model simulations.
