# Supplementary material for: Wildfire response to changing daily temperature extremes in California’s Sierra Nevada
Source: Sci Adv. 2021 Nov 17;7(47):eabe6417. doi: 10.1126/sciadv.abe6417 (PMC8597996; doi:10.1126/sciadv.abe6417)
Supplement: Supplementary file 1 — Figs. S1 to S8 Table S1 [file sciadv.abe6417_sm.pdf]

Science Advances
AAAS

Supplementary Materials for

Wildfire response to changing daily temperature extremes in California's Sierra Nevada

Aurora A. Gutierrez*, Stijn Hantson, Baird Langenbrunner, Bin Chen, Yufang Jin, Michael L. Goulden, James T. Randerson

*Corresponding author. Email: auroraag@uci.edu

Published 17 November 2021, *Sci. Adv.* **7**, eabe6417 (2021)
DOI: 10.1126/sciadv.abe6417

This PDF file includes:

Figs. S1 to S8
Table S1

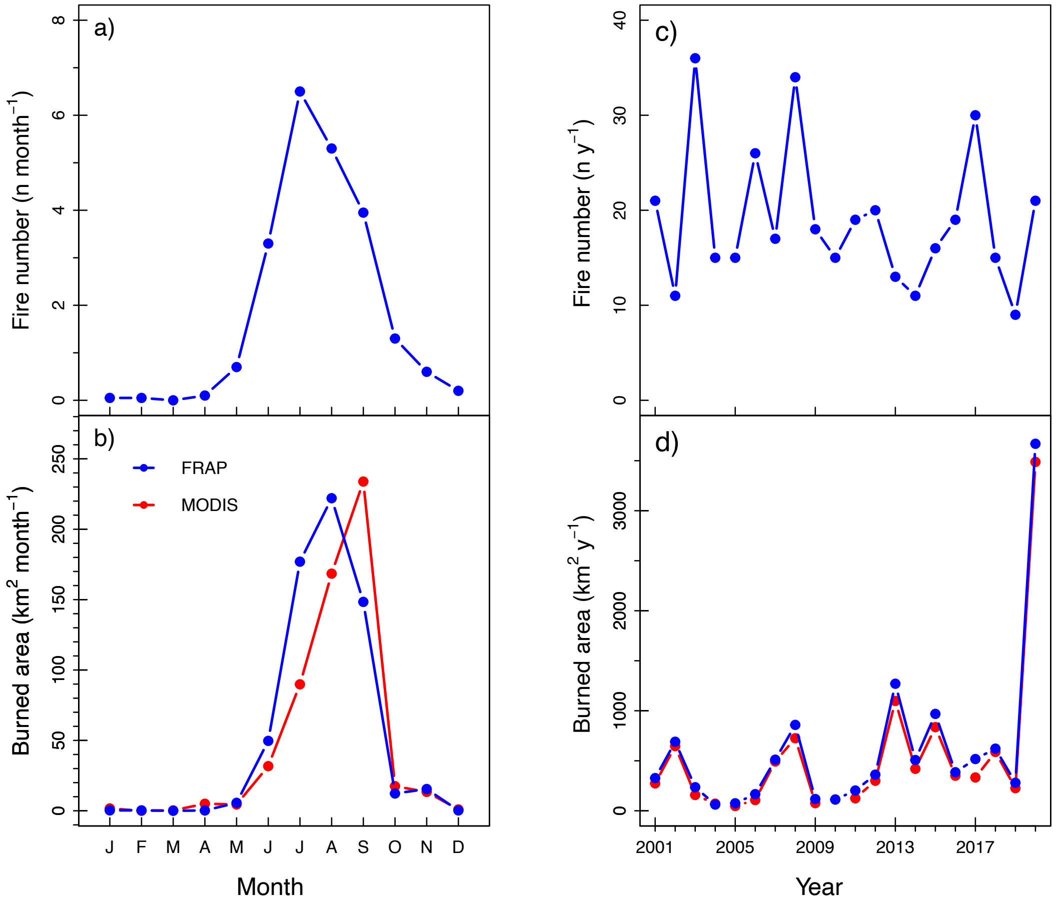

Fig. S1. Annual mean cycle of fire number and burned area (panel a and b) in the Sierra Nevada level 3 ecoregion derived from the FRAP fire perimeter database and the MODIS daily burned area product during 2001-2020. FRAP burned area for the entire fire duration is assigned to the reported discovery date (first day of detection) and therefore differs in an expected way from annual mean cycle derived from daily-level MODIS burned area observations. Annual time series (panels c and d) of fire number and burned area during summer (June-September) for the Sierra Nevada level 3 ecoregion.

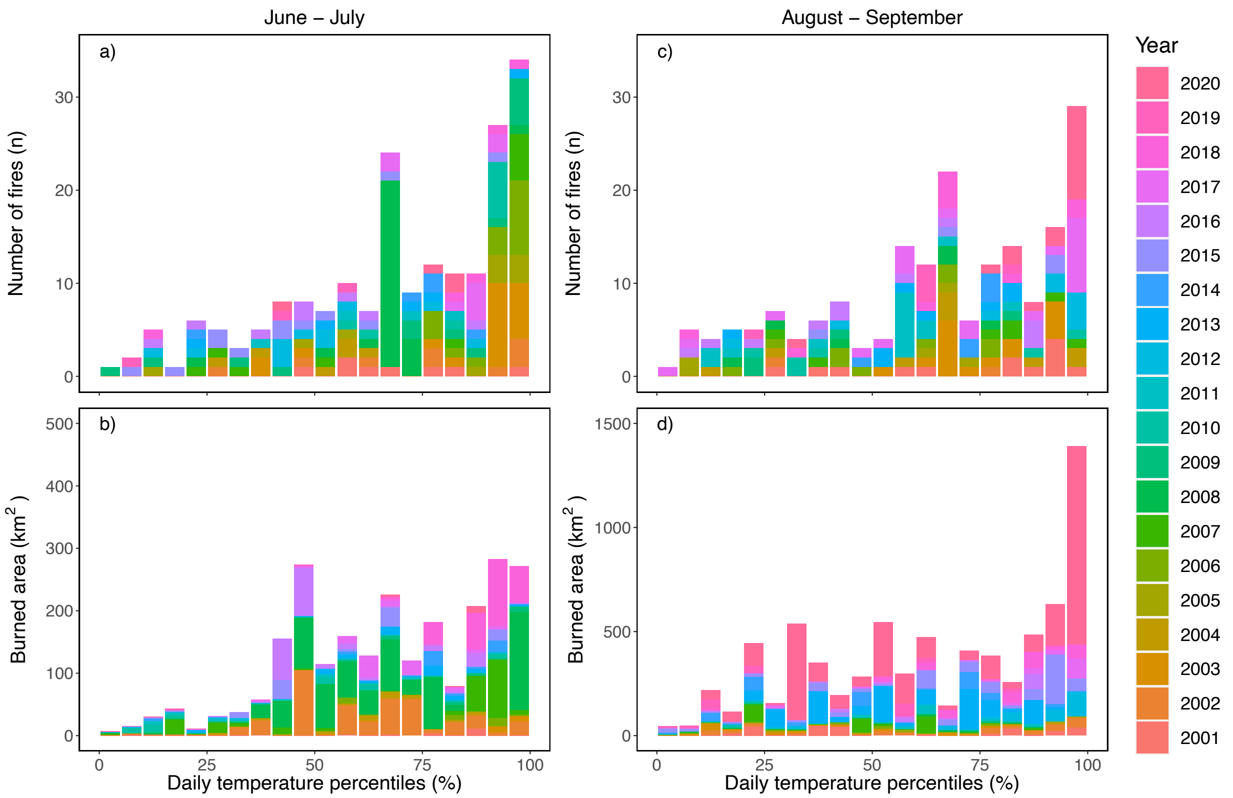

Fig. S2. Fire activity (number of fires and burned area) as a function of percentiles of daily temperature during 2001-2020 in the Sierra Nevada level 3 ecoregion. Panels a and b show fire number and burned area for the June-July period, while panels c and d show fire number and burned area for the August-September period. The color bar shows the year of fire occurrence or burned area.

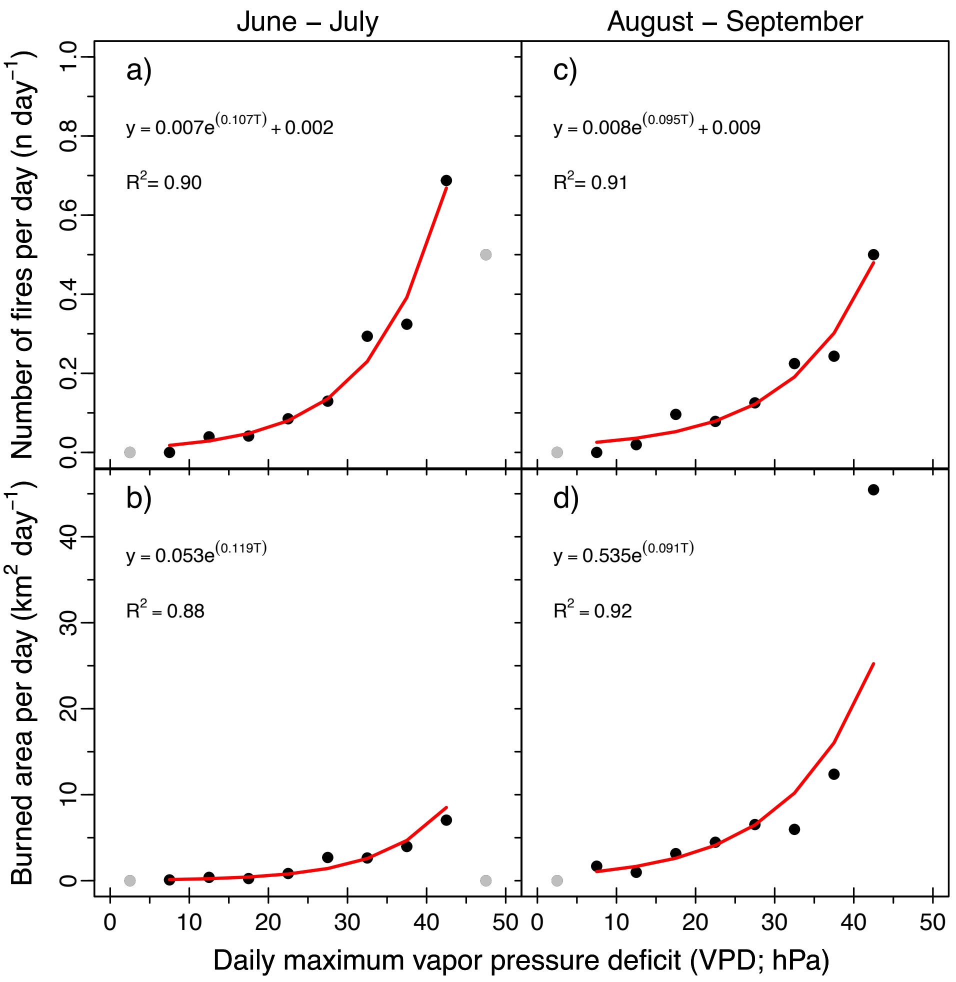

Fig. S3. Probability of the number of fires or burned area as a function of daily maximum VPD for June-July (a, b) and August-September (c, d) periods in the Sierra Nevada ecoregion. Each point indicates the number of fires or burned area for a day within each 5 hPa VPD interval. All of the panels show significant increasing trends in fire activity as a function of VPD with p-values less than 0.01. VPD intervals that capture less than 10 of the total number of summer days during 2001-2020 are shown in gray and were not used in model fitting. This figure is analogous to the bottom panels of Fig. 2 and Fig. 3, replacing daily mean temperature with daily maximum VPD.

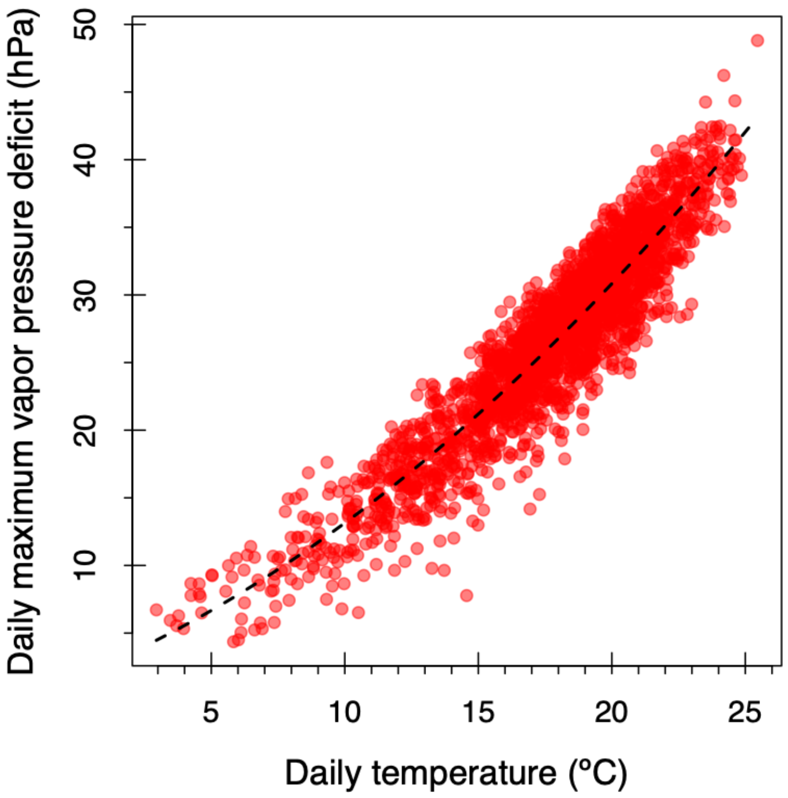

Fig. S4. Daily maximum VPD as a function of daily mean temperature (ºC) for summer observations from the Sierra Nevada level 3 ecoregion during 2001 to 2020. All of the observations are from PRISM.

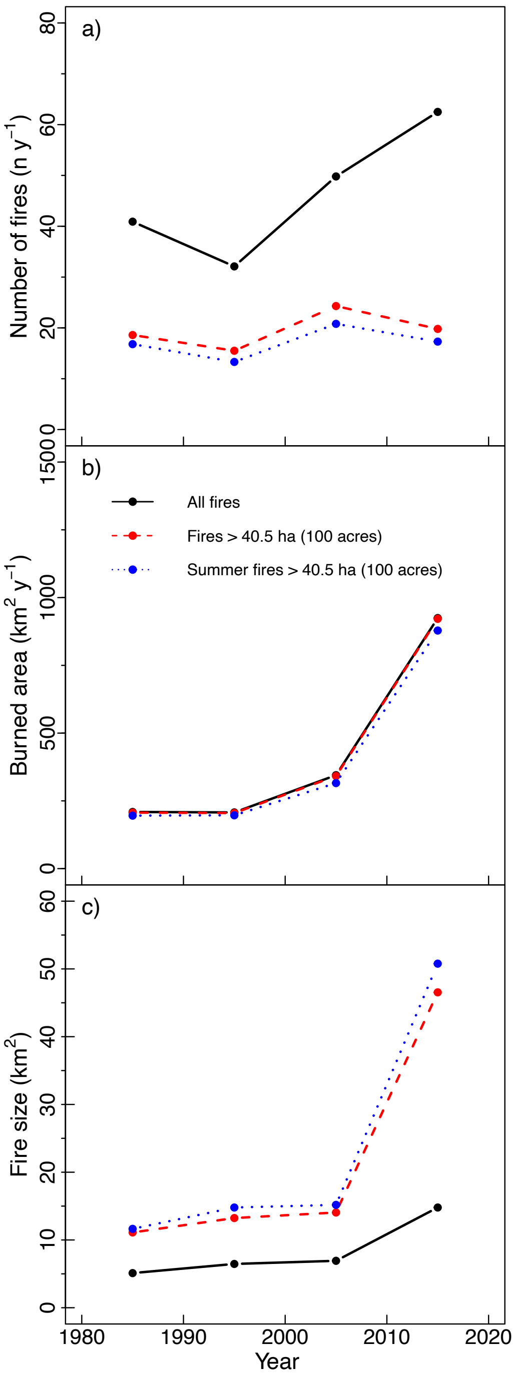

Fig. S5. Time series of decadal mean number of fires (a), burned area (b), and fire size (c) during 1981-2020 in the Sierra Nevada ecoregion. All observations are from FRAP.

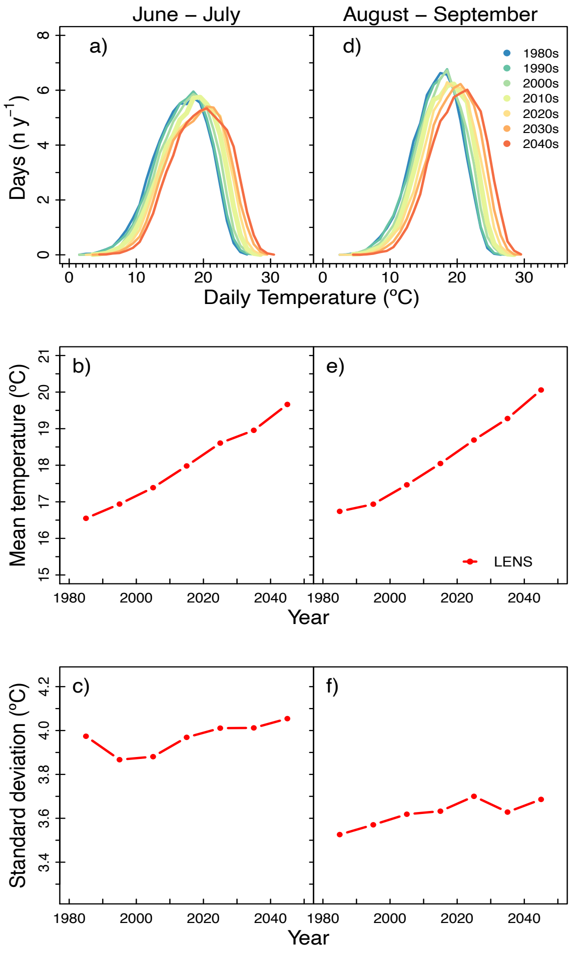

Fig. S6. Changes in summer daily temperature from the Community Earth System Model version 1 Large Ensemble (LENS) for the Sierra Nevada level 3 ecoregion. Model estimates for the June-July summer interval are shown in panels a, b and c, and for the August-September interval in panels d, e, and f. The top row (a, d) shows the mean probability distribution function (PDF) for daily temperature averaged by decade averaged across the 40 LENS ensemble members. The middle row (b, e) shows the mean temperature during each summer interval. The bottom row (c, f) shows the standard deviation of daily temperature during each summer interval. The standard deviations shown in panels c and f were computed in the following way. First, we computed the daily temperature standard deviation for each simulation, pooling all the data together for that decade. In a second step we then took the average of these standard deviations across the 40 different ensemble members. The analysis in the bottom row indicates that daily summer temperature within each climate simulation is becoming more variable with time. The increasing standard deviation of daily temperature (c, d), along with divergence of individual model ensemble members from internal climate variability, explains the widening of the PDFs shown in panels a and c.

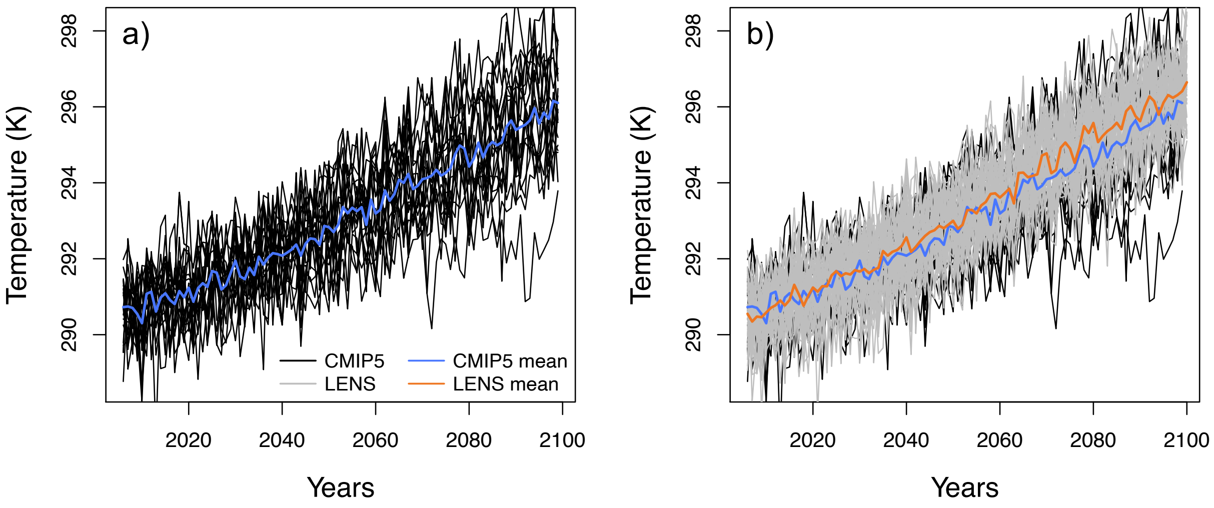

Fig. S7. Comparison of the trends and variability between CESM1 LENS and other CMIP5 model simulations for RCP85 processed using MACA for the period of 2006-2100 for the Sierra Nevada level 3 ecoregion. Panel a shows the bias corrected summer temperature time series (June-September) for 20 CMIP5 models over the Sierra Nevada level 3 region processed using MACA (59). Each simulation is shown in black and the multi-model mean is shown in blue. Panel b shows the mean trend from LENS (red) and year variability among ensemble members (gray) overlain on the mean and variability from the CMIP5 models.

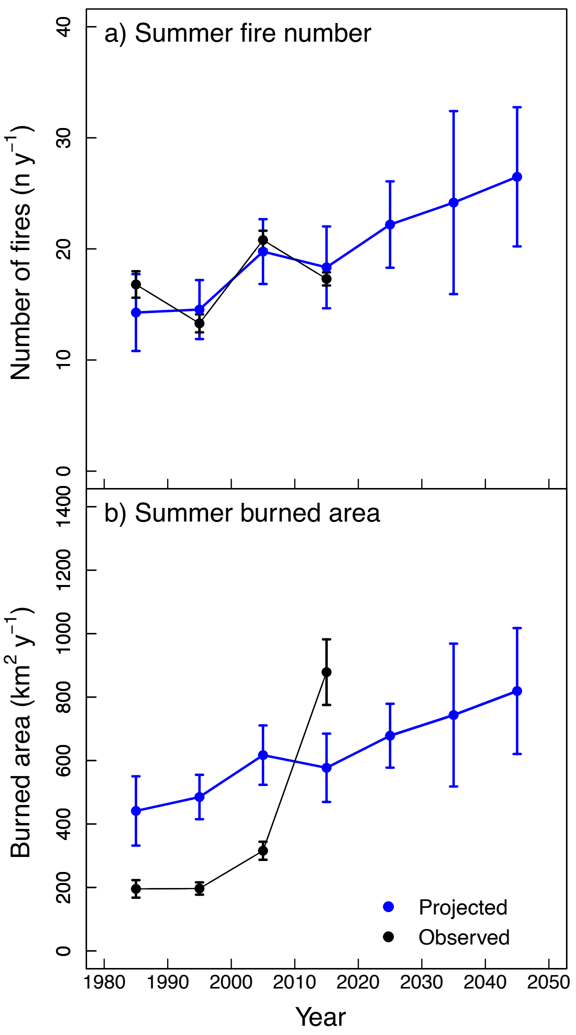

Fig. S8. Projected future changes in fire number (a) and burned area (b) derived using contemporary relationships between daily summer VPD and wildfire activity. Observations of summer fire number and burned area from the FRAP dataset are shown in black. The model estimates in blue show the impact of changing maximum daily VPD on fire activity using the relationships derived from contemporary fire observations. The uncertainties in the future projections were estimated by combining uncertainties from the form of the VPD – wildfire relationships (obtained using a jackknifing resampling approach) with variation in future projections in VPD obtained from different LENS ensemble members. The uncertainty bars represent ± 1 standard deviation. Projections of the impact of changing VPD on fire activity from the 1980s through 2010s were obtained using vapor pressure deficit observations from PRISM. Future projections of the impact of changing maximum daily VPD on fire activity from the 2010s through the 2040s were obtained using earth system model simulations from CESM1 LENS.

Fire variable	Linear Fit (2 parameters)		Exponential Fit (2 parameters)	
	R^2	P-value	R^2	P-value
Jun.-Jul. number of fires	0.71	1.4e-05	0.88	1.2e-08
Jun.-Jul. burned area	0.73	7.8e-06	0.88	1.4e-08
Aug.-Sept. number of fires	0.51	0.0011	0.51	0.0011
Aug.-Sept. burned area	0.57	0.00047	0.82	7.9e-07

Table S1. Goodness of fit estimates (R^2 and P values) linear and log-transformed fire variables as a function of daily temperature shown in Figures 2 and 3. All of the linear and log-transformed regression models had a p value less than 0.01.
